# Effects of internet-based health education on patients with acute intermittent porphyria

**DOI:** 10.1186/s13023-024-03440-2

**Published:** 2024-11-15

**Authors:** Lanlan Zhao, Yuhan Liu, Jie Li, Pei Li, Xin Zhao, Songyun Zhang

**Affiliations:** 1 Department of Endocrinology & Rare Diseases, The Second Hospital of Hebei Medical University, Shijiazhuang, Hebei 050000 China; 2 Department of Nephrology, The Second Hospital of Hebei Medical University, Shijiazhuang, Hebei 050000 China; 3 Hebei Key Laboratory of Rare Diseases, Shijiazhuang, Hebei 050000 China

**Keywords:** Health education, Acute intermittent porphyria, Mental health, Quality of life, Internet-based

## Abstract

**Background:**

Acute intermittent porphyria (AIP) is a rare genetic metabolic disorder characterized by acute attacks of neurovisceral symptoms. This disease not only poses a threat to patients’ physical and mental well-being, but its frequent acute attacks also have a profound impact on patients’ mental state and overall quality of life (QoL).

**Objective:**

This study aimed to explore the impact of internet-based health education on the acute attacks, mental health, and QoL of patients with AIP.

**Methods:**

This study employed a pre-post comparison design, recruiting 52 patients diagnosed with AIP and treated at the Second Hospital of Hebei Medical University between September 2021 and May 2023 as the subjects of investigation. All participants underwent a 12-month internet-based health education intervention. Quantitative assessments of the intervention’s efficacy in reducing acute attacks, enhancing mental health status, and improving QoL among AIP patients were conducted using various instruments, including measures of acute episode frequency and severity, the Depression Anxiety Stress Scales-21 (DASS-21), the Positive and Negative Affect Schedule (PANAS), and the MOS 36-Item Short Form Health Survey (SF-36). Data were collected at two distinct time points: pre- and post-health education interventions, which were then subjected to comparative analysis.

**Results:**

Compared to pre-health education, the frequency of acute attacks among patients with AIP significantly decreased post-health education intervention (*p* < 0.05). Furthermore, notable improvements were observed in the severity of acute attacks, PANAS scores, DASS-21 scores, and SF-36 scores (*p* < 0.05).

**Conclusions:**

This study validated that health education effectively reduced the frequency and severity of acute attacks in AIP patients while enhancing their mental health status and quality of life. Internet-based health education emerges as a practical and productive strategy for AIP patients.

## Introduction

Acute Intermittent Porphyria (AIP) is a rare autosomal dominant inherited metabolic disorder (OMIM:176000) [1, 2] and is often precipitated by factors such as infections, medications, and negative emotions. Its clinical manifestations, characterized by severe neurovisceral symptoms, can pose a life-threatening condition [3, 4].

Apart from physical impairments, AIP poses a significant threat to patients’ mental health as well. Research has demonstrated that approximately 50–60% of AIP patients experience emotional disorders such as anxiety and depression [5, 6], leading to a general decline in their QOL [7, 8]. As an effective intervention, health education can significantly enhance patients’ understanding of the disease and their self-management abilities. It aids patients in proactively avoiding triggering factors, alleviating negative emotions, improving mental health status, and ultimately enhancing their QOL [9, 10]. In recent years, health education has been extensively applied in the prevention and management of chronic diseases such as diabetes and hypertension, yielding notable outcomes [11]. However, research on the influence of health education on patients with AIP remains limited. This study, focusing on patients with AIP in our hospital, employed a self-controlled before-and-after design to investigate the impact of internet-based health education on the frequency and severity of acute attacks, patients’ mental health, and QOL. The findings aimed to provide insights for optimizing clinical strategies to prevent acute attacks of AIP.

## Materials and methods

### Participant recruitment and criteria

This study included 52 patients with AIP who were admitted to the Second Hospital of Hebei Medical University from September 2021 to May 2023. The study was approved by the Ethics Committee of the Second Hospital of Hebei Medical University (Approval No. 2023-R597) and adhered to the Declaration of Helsinki. All patients were informed of the purpose and significance of the study and provided written informed consent.

Inclusion criteria were as follows: (1) Diagnosis of AIP confirmed through genetic analysis, with a positive urine porphobilinogen (PBG) test. (2) Intact consciousness, normal cognitive functioning, and the absence of any communication barriers. (3) Voluntary participation with complete and accurate information provided. (4) The ability of patients or their caregivers to utilize smartphones and social media applications such as WeChat.

Exclusion criteria were as follows: (1) A history of alcohol or drug addiction. (2) The presence of severe internal medical conditions, such as cancer or organ failure involving the heart, liver, lungs, or kidneys. (3) Participation in any other concurrent clinical research studies.

### Study design and procedures

The health education intervention in this study was organized and executed by the Multi-disciplinary Treatment (MDT) health education team at the Second Hospital of Hebei Medical University, spanning 12 months. The content primarily encompassed disease knowledge education, mental health guidance, and WeChat-based doctor-patient interactions.

1. Health education team: The health education team comprised one chief physician, one associate chief physician, three attending physicians, three resident physicians, and a psychological counselor. Each member possessed experience in managing AIP patients and has undergone professional training. The training program covered a wide range of topics and skills, including but not limited to psychology training, health education knowledge and skills training, communication skills training, training in interdisciplinary cooperation concepts, learning of AIP’s latest clinical guidelines, diagnostic techniques, and treatment strategies, professional development and continuing education (participation in academic conferences, seminars, online courses, etc.).
2. Development and implementation of health education content: Through a questionnaire survey, specific patient needs were gathered, upon which the health education team collaboratively formulated a scientific health education plan, drawing upon a literature review and past experiences. This health education program comprises two primary components:

①Disease knowledge lectures: Every two months, the health education team organizes approximately 2 to 3 h of online lectures (typically commencing at 7:00 PM) via Tencent Meeting. Patients with AIP and their families accessed these sessions by scanning pre-distributed QR codes. The lectures centered on two primary themes: the significance of health education and an overview of AIP disease. Each lecture concluded with a question-and-answer segment, during which participants had the opportunity to consult experts from various disciplines within the porphyrin collaboration team for tailored responses. These lectures aimed to elucidate the etiology, pathogenesis, clinical manifestations, treatment, and prevention measures of AIP. Furthermore, the lectures emphasized the reinforcement of health education, enhancing patients’ understanding of its importance. Through imparting knowledge, patients were guided to modify and discard unhealthy behavioral patterns, ultimately improving treatment adherence and self-management capabilities.

② Psychological counseling: Every two months, approximately 1–2 h of mental health guidance was delivered via Tencent Meeting. Initially, patients were assisted in understanding the underlying causes of negative emotions, guided to articulate their feelings, and empowered to develop confidence in combating their illness. Subsequently, they were encouraged to regulate their emotional states and instructed on mental relaxation techniques, including attention shifting and emotional regulation methods, to facilitate self-regulation. Ultimately, regular assessments of the mental health status of AIP patients were conducted (every two months), and individualized guidance and treatment were provided offline for those who experienced mental health issues.

(3) Follow-up and feedback: An interactive WeChat platform was established to cater to the follow-up and guidance needs of AIP patients. This platform facilitated patients’ understanding of common triggers for acute attacks, such as medications, infections, and emotional stress. Additionally, patients were provided with strategies to avoid these triggers, methods to recognize premonitory and early symptoms of AIP, and effective means of managing them. Through the WeChat platform, periodic dissemination of AIP-related knowledgewas undertaken. Team members were assigned to specific patients, conducting periodic inquiries into their conditions, guiding follow-up, promptly addressing patients’ queries, and offering tailored guidance.

### Quantitative assessment

Demographic information of AIP patients, including gender, age, body mass index (BMI), education level, marital status, and employment status, was collected. Data about the annual frequency of acute attacks, the severity of attacks, Positive and Negative Affect Scale (PANAS), Depression Anxiety and Stress Scale (DASS-21), and the MOS 36-item Short-Form Health Survey (SF-36) scores were gathered both before and 12 months after the implementation of health education.

1. Frequency and Severity of acute attacks: Due to the current lack of capabilities for the quantitative measurement of aminolevulinic acid (ALA) and PBG in China, a questionnaire was designed to assess the frequency and severity of AIP attacks based on descriptions in existing literature and clinical practice experience. Acute attacks were defined as acute symptoms involving pain, autonomic and peripheral neuropathy, and central nervous system involvement, which required hospitalization, emergency treatment, intravenous heme infusion, or administration of hypertonic glucose, either intravenously or orally [12–14]. Severe acute attacks were characterized by one or more of the following : severe hyponatremia (serum sodium < 125 mmol/L), peripheral neuropathy (limb paralysis), central nervous system involvement (epilepsy, dyspnea, and consciousness disorders), and arrhythmias [12].
2. Mental Health assessment: Given the comprehensiveness, wide applicability, specialized design, ease of use, and high reliability and validity of the PANAS and DASS-21 in emotional assessment, this study has decided to combine these two scales to conduct an in-depth evaluation of individuals’ emotional states.

① PANAS: The PANAS was originally developed by Watson and Clark in 1988 and consists of two subscales [15]. Specifically, the Positive Affect (PA) subscale encompassed ten items that describe positive emotions such as “interested” and “excited,” while the Negative Affect (NA) subscale comprises ten items that capture negative emotions like “distressed” and “guilty.” Participants are required to carefully read each item and rate it according to their current emotional state. Typically, ratings were conducted using a Likert 5-point scale, ranging from 1 (very slightly or not at all) to 5 (extremely). The total score for each subscale ranges from 10 to 50, with higher scores indicating a greater intensity of experienced positive or negative emotions.

② DASS-21: The DASS-21 was initially developed by British psychologist Lovibond in 1995 and later revised and refined by Anthony. The scale consisted of three subscales measuring negative emotional states: depression, anxiety, and stress. Each subscale contained seven items, resulting in a total of 21 items [16]. A 4-point Likert scale was used for scoring, ranging from 0 (does not apply) to 3 (always applies). The total score for each subscale ranges from 0 to 21, with higher scores indicating greater severity of depression, anxiety, or stress experienced by the patient.

(3) QOL assessment: The SF-36, a generic measurement scale, was developed by the Medical Outcomes Study (MOS) in the United States. It was primarily designed to assess eight dimensions of health-related QoL [17]: Physical Function (PF), Role Physical (RP), Body Pain (BP), General Health (GH), Vitality (VT), Social Function (SF), Role Emotional (RE), and Mental Health (MH). The SF-36 consisted of 36 items, with its eight dimensions categorized into two domains: physical health (including PF, RP, BP, and GH) and mental health (encompassing VT, SF, RE, and MH). These domains were subsequently summarized as the physical component summary (PCS) and the mental component summary (MCS), offering a comprehensive evaluation of both physical and mental well-being.

### Statistical analysis

All statistical analyses were performed using SPSS version 25.0. Continuous variables with a normal distribution were summarized with mean ± standard deviation (SD), and comparisons between groups were conducted using paired t-tests. For continuous variables with a skewed distribution, data were presented as the median and interquartile range (P25, P75). Group comparisons were performed using the paired Wilcoxon signed-rank test. Categorical variables were expressed as the number of cases (percentage), and comparisons between groups were conducted using the Chi-squared or Fisher’s exact test. A *P* value < 0.05 was considered statistically significant.

## Results

### Demographic characteristics

**Fig. 1:**
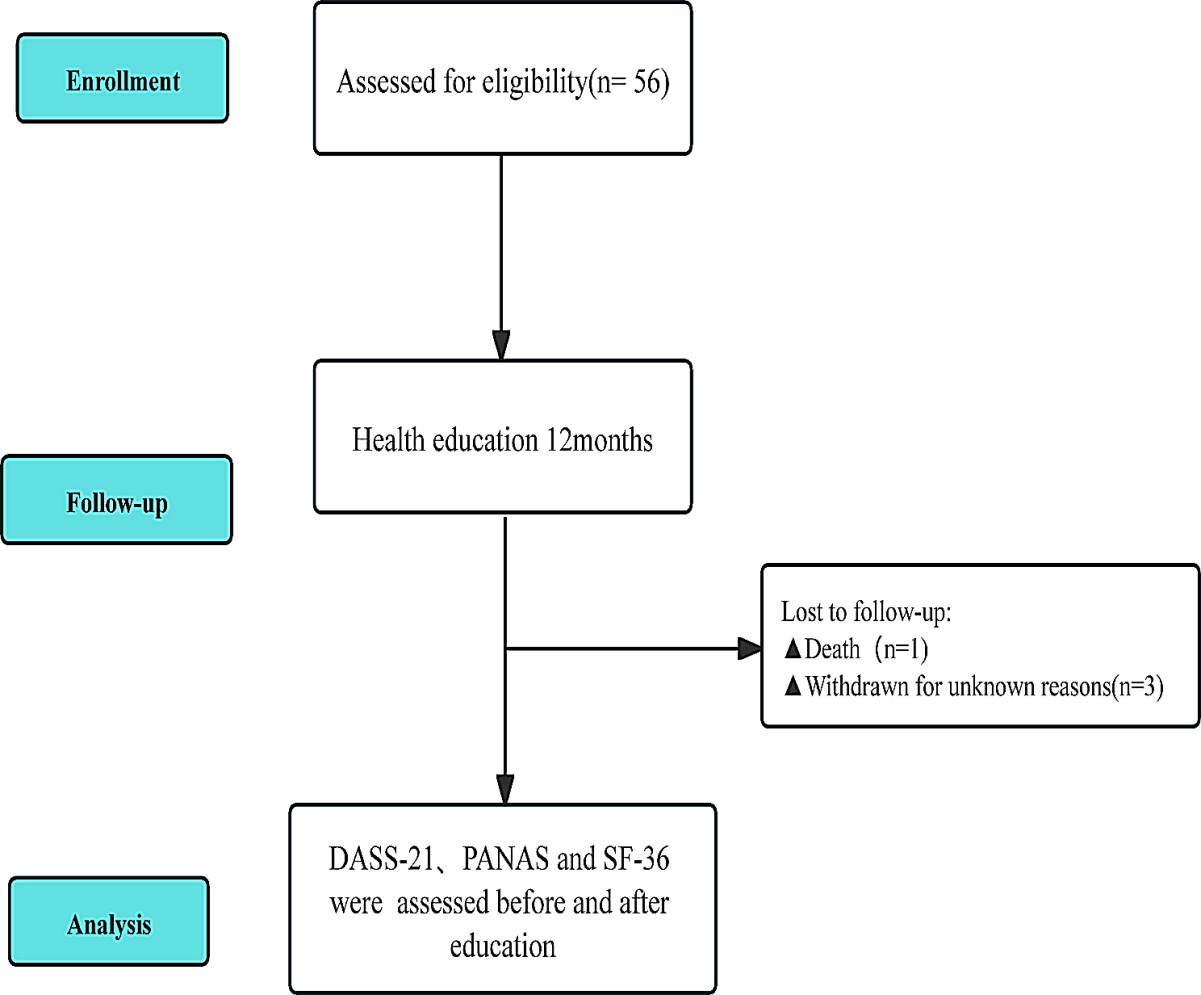
Study flowchart

A total of 56 AIP patients were initially selected for this study. However, one patient passed away during the health education period, and three others withdrew from the study for unknown reasons. Ultimately, 52 AIP patients were included in the analysis, as shown in the study flowchart (Fig. 1). Among the 52 patients, two were male, and fifty were female, with ages ranging from 17 to 39 years and a mean age of 27.37 ± 6.36 years. BMI ranged from 16.14 to 28.91 kg/m², with a mean BMI of 21.53 ± 2.84 kg/m². Approximately 21 patients (40.38%) were unemployed, as detailed in Table 1.

**Table 1:** Demographic characteristics of AIP patients

Gender, *N* (%)	
Male	2(3.85%)
Female	50(96.15%)
Age range (years)	[17–39]
Age (mean ± SD)	27.37 ± 6.36
BMI range (years)	[16.14–28.91]
BMI (mean ± SD)	21.53 ± 2.84
Education, N (%)	
Primary school	2(3.85%)
Junior high school	10(19.23%)
High school (Technical secondary School)	9(17.31%)
College(Junior college)	29(55.77%)
Postgraduate	2(3.85%)
Marital status, N (%)	
Married	26(50%)
Divorced or separated	2(3.85%)
Single	24(46.15%)
Employment, N (%)	
Full-time	22(42.31%)
Unemployed	21(40.38%)
Student	9(17.31%)

### Frequency and severity of acute attacks before and after health education

Compared to before health education, the frequency of AIP acute attacks significantly decreased after the intervention (*P* < 0.05). Specifically, the incidence of frequent acute attacks (≥ 4 times/year) dropped markedly, from 55.8 to 23.1%. Additionally, the number of severe AIPattacks significantly declined after the intervention (*P* < 0.05). The proportion of patients experiencing three or more attacks decreased substantially, from 17.3 to 0%. Further details are provided in Table 2.

**Table 2:** Comparison of the frequency and severity of acute attacks before and after health education

	Before intervention	After intervention	*P*
Number of attacks in the last 1 years, N (%)			0.005^**^
0	4(7.7%)	12(23.1%)	
1–3	19(36.5%)	28(53.8%)	
4–6	16(30.8%)	7(13.5%)	
7–9	9(17.3%)	5(9.6%)	
≥ 10	4(7.7%)	0(0%)	
Number of severe attacks in the last 1 years, N (%)			0.000^***^
0	15(28.8%)	36(69.2%)	
1	16(30.8%)	9(17.3%)	
2	12(23.1%)	7(13.5%)	
≥ 3	10(17.3%)	0(0%)	

* *P* < 0.05, ** *P* < 0.01, ***P < 0.001

### Mental health status assessment before and after health education

There was a statistically significant difference in PANAS scores before and after the health education intervention (*P* < 0.05). Specifically, the PA subscale score increased significantly (28.46 ± 6.52 vs. 26.55 ± 7.02, *P* < 0.05), while no significant difference was observed in the NA subscale score (*P* > 0.05). Additionally, compared to pre-education levels, the DASS-21 subscale scores for anxiety (3.00 vs. 4.00, *P* < 0.05) and stress (3.67 ± 3.05 vs. 5.02 ± 4.08, *P* < 0.05) both showed significant reductions after the health education intervention (*P* < 0.05), as detailed in Table 3.

**Table 3:** Comparison of PANAS and DASS-21 scores before and after education

		Before intervention	After intervention	*P*
PANAS	PA	26.55 ± 7.02	28.46 ± 6.52	0.032*
	NA	22.98 ± 6.27	23.13 ± 5.85	0.862
DASS−21	Depression	3.50(1.00,6.00)	2.00(0,5.75)	0.111
	Anxiety	4.00(2.00,6.00)	3.00(1.00,4.75)	0.034*
	Stress	5.02 ± 4.08	3.67 ± 3.05	0.006**

* *P* < 0.05, ** *P* < 0.01, ***P < 0.001

### QoL assessment before and after health education

Compared to pre-education levels, the PCS score significantly increased after health education (289.18 ± 75.70 vs. 241.21 ± 97.76, *P* < 0.05). Before the intervention, the mean PF score was 78.27, which increased to 87.79 after the intervention (*P* < 0.05), indicating that health education effectively improved PF in AIP patients. Furthermore, post-intervention scores for RP (75.00 vs. 25.00, *P* < 0.05) and BP (81.50 vs. 75.50, *P* < 0.05) both showed significant improvements. However, no statistically significant difference was observed in the MCS score before and after health education (298.83 vs. 275.33, *P* > 0.05). Notably, the MH score increased significantly after the intervention (66.62 ± 13.56 vs. 61.62 ± 16.71, *P* < 0.05), as detailed in Table 4.

**Table 4:** Comparison of SF-36scores before and after health education

	Before intervention	After intervention	*P*
PCS	241.21 ± 97.76	289.18 ± 75.70	0.000^***^
PF	90.00(70.00,95.00)	90.00(80.00,98.75)	0.021^*^
RP	25.00(0,100.00)	75.00(25.00,100.00)	0.001^***^
BP	75.50(38.75,100.00)	81.50(69.50,100.00)	0.001^***^
GH	52.50(40.00,63.75)	55.00(40.00,70.00)	0.939
MCS	275.33(199.25,329.88)	298.83(222.25,332.50)	0.261
VT	60.00(45.00,78.75)	65.00(45.00,80.00)	0.486
SF	75.00(62.50,87.50)	75.00(75.00,87.50)	0.132
RE	100.00(33.33,100.00)	100.00(33.33,100.00)	0.347
MH	61.62 ± 16.71	66.62 ± 13.56	0.039^*^

* *P* < 0.05, ** *P* < 0.01, ***P < 0.001

## Discussion

The clinical manifestations of AIP are diverse, with life-threatening acute attacks being the primary feature. These attacks can affect multiple organs and systems throughout the body, severely impacting both the physical and mental health of patients, as well as their QoL. Research has shown that anxiety, depression, and psychological stress are not only common triggers for AIP acute attacks but also constitute a significant component of the overall symptomatology of AIP [18]. In 2019, a single-center study in Germany found that approximately 61% of AIP patients from 57 families suffered from depression, and about 52% experienced anxiety [19]. In 2022, a global study involving 92 patients with acute hepatic porphyria (AHP) from six countries reported that approximately 58.7% of patients experienced moderate to severe depression, while 48.9% had moderate to severe anxiety [20]. A study on AHP conducted in Brazil revealed that approximately 82 patients (78%) with AHP experienced emotional disturbances, sleep disorders, and fatigue. Among them, 73 patients (69.5%) suffered from anxiety, and 47 patients (44.7%) reported a lack of motivation [21]. In addition, a study by Yutaka Horie and colleagues indicated that among 391 Japanese AHP patients, approximately 42 (10.7%) had mood disorders, and around 85 (21.7%) suffered from neurotic, stress-related, and somatoform disorders. The prevalence of anxiety and depression was 11.3% and 9%, respectively [22]. QoL impairments were prevalent across various porphyria subtypes, and it was particularly pronounced in AHP due to the frequent acute attacks. The disease course can last from several months to years, posing a serious lifelong threat to the health of affected patients [8]. Millward et al. used the European Quality of Life Questionnaire (EuroQoL) to assess the health-related QoL in AIP patients. The results showed a significant reduction in QoL among porphyria patients, with the decline being particularly pronounced in those with AIP [23]. A cohort study conducted in Germany involving 57 AIP patients found that QoL was generally impaired due to paralysis, fear of acute attacks, fatigue, or weakness. The average QoL score was 5 out of 10 [5]. In addition, Yang et al. assessed the QoL in 27 female AIP patients using the SF-36 scale. The results revealed significantly lower SF-36 scores, particularly in the physical health dimension [24]. Health education is a carefully planned, organized, and evaluated educational intervention aimed at encouraging patients to adopt behaviors and lifestyles that promote health. Its primary objectives include optimizing treatment adherence, accelerating disease recovery, and ultimately improving patients’ QoL [25]. Given the indispensable role health education has played in the prevention and management of chronic diseases in recent years and its notable success, we hypothesize that health education could be an effective strategy for enhancing AIP patients’ awareness and self-management abilities, reducing the risk of acute attacks, and improving their mental health and QoL. However, with the rapid development of information technology, traditional health education faces challenges such as a lack of continuity and limited accessibility in disseminating disease-related knowledge, making it difficult to meet the information needs of patients and their families [26]. In response to these challenges, internet-based health education has emerged and gradually demonstrated its unique advantages and value in clinical disease management.

The results of this study demonstrated that health education significantly reduced the frequency of acute attacks and lessened the severity of such attacks in AIP patients (*P* < 0.01). Internet-based health education has demonstrated several positive impacts. First, it provided patients with comprehensive knowledge about AIP, enhancing their understanding of the disease, its triggers, and effective treatment strategies. Second, this educational approach improved patients’ self-management skills and encouraged active participation in disease management. Enabling patients to identify the triggers and early symptoms of acute attacks motivated them to take proactive steps to avoid these triggers and initiate timely treatments, such as increasing sugar intake at the onset of prodromal symptoms. These interventions aided in preventing acute attacks and reducing the progression of symptoms. Additionally, the development of an interactive WeChat platform ensured continuity and consistency in the follow-up care of AIP patients while also providing personalized and precise guidance. This greatly enhanced the timeliness of support, ensuring patients receive the necessary professional assistance and guidance promptly. Anxiety, depression, and stress are not only common triggers of AIP acute attacks but also form an integral part of their clinical symptoms. The results of this study demonstrated the expected psychological benefits of health education. After receiving health education, significant improvements were observed in the PA scores of AIP patients (28.46 ± 6.52 vs. 26.55 ± 7.02, *P* < 0.05), with marked reductions in stress levels (3.67 ± 3.05 vs. 5.02 ± 4.08, *P* < 0.05) and anxiety levels (3.00 vs. 2.00, *P* < 0.05). This significant change highlights the positive role of health education in promoting emotional stabilization and optimizing mental health. By deepening patients’ understanding and awareness of AIP, a more accurate and scientific perception of the disease can be fostered. On this basis, mental health education, as an important supplement, significantly enhanced patients’ sense of self-efficacy. This empowerment enabled them to face disease-related challenges with greater confidence and composure, promoting the regulation of positive emotions and further strengthening their emotional health and psychological stability.

The findings demonstrated that internet-based health education had a positive impact on the QoL in AIP patients, particularly in the area of physical health (289.18 ± 75.70 vs. 241.21 ± 97.76, *P* < 0.01). Significant improvements were primarily observed in the PF, RP, and BP dimensions compared to pre-education scores (*P* < 0.05). This finding further confirmed that regular health education enabled patients to actively avoid potential triggers of acute attacks, thereby preventing AIP acute attacks. Additionally, when prodromal symptoms appear, effective measures such as increased glucose intake can be taken to avoid the progression of symptoms. As specific AIP medications such as Givosiran and Hemin are not yet available in China, their preventive use is not feasible. This has made increased glucose intake the primary treatment for acute AIP attacks in the country. Although no significant changes were observed in the MCS scores of the SF-36 scale before and after education, a significant improvement was noted in the MH dimension following education (66.62 ± 13.56 vs. 61.62 ± 16.71, *P* < 0.05), indicating a clear improvement in mental health. These results were consistent with the findings reported in the third section of this study. In conclusion, it was determined that the pivotal mechanism by which health education improved the QoL in AIP patients was its significant enhancement of patients’ self-management abilities. This enhancement in ability encouraged patients to take a more active role in various aspects of health decision-making and daily management, fostering a more proactive and autonomous approach to health management. This shift not only significantly improved treatment adherence, enabling patients to strictly follow medical advice and effectively implement treatment plans also contributed to better control of acute attacks, reducing the impact of the disease on their lives. Ultimately, this series of positive cascading effects collectively contributed to a significant improvement in the QoL for AIP patients, underscoring the central role and profound impact of health education in the management of chronic diseases.

Our study presented several important implications. First, AIP patients, as a vulnerable population, had particularly challenging care needs. This research preliminarily explored the impact of health education on AIP, revealing that it not only reduced the frequency and severity of acute attacks but also significantly improved the psychological health and QoL of AIP patients. This finding offered a practical and effective approach to preventing acute attacks and improving the overall health status of AIP patients, holding significant clinical value. Additionally, the health education implemented in this study was delivered through an online platform, yielding notable results. This method ensured that health education information was provided more effectively, conveniently, and broadly. Through the Internet, patients could access relevant health knowledge anytime and anywhere, significantly enhancing the utilization of AIP-related information. This innovative model not only demonstrates the application of technology in the medical field but also provides new ideas and directions for the future development of health education.

### Limitations

This study has certain limitations that should be addressed in future research. First, as this study was based on self-comparison without a control group, it was challenging to eliminate the influence of external events or factors that may have occurred during the research period. This limitation could introduce bias when evaluating the actual effects of the health education intervention. Therefore, future studies should include one or more control groups to more accurately assess the effectiveness of health education measures. Second, the scales used in this study were self-report questionnaires, which might introduce a degree of subjectivity. Future research could incorporate additional assessment tools for a more comprehensive evaluation. Third, this study was a single-center, retrospective analysis with a limited number of cases. Multi-center, prospective studies are needed to further validate these findings.

## Conclusions

This study indicated that internet-based health education effectively reduced the frequency and severity of acute attacks in AIP patients, significantly improved their mental health, and enhanced their QoL. These findings hold important clinical significance for broader application.

## Data Availability

The datasets used and/or analysed during the current study are available from the corresponding author .

## Abbreviations

AIP: Acute intermittent porphyria
AHP: Acute hepatic porphyria
ALA: Aminolevulinic acid
BP: Body Pain
BMI: Body mass index
DASS-21: Depression Anxiety Stress Scales-21
EuroQoL: European Quality of Life Questionnaire
GH: General Health
MDT: Multidisciplinary Teamwork
MH: Mental health
MOS: Medical Outcomes Study
MCS: Mental component summary
NA: Negative Affect
PA: Positive Affect
PF: Physical Function
PBG: Porphobilinogen
PANAS: Positive and Negative Affect Schedule
PCS: Physical component summary
QoL: Quality of life
RP: Role Physical
RE: Role Emotional
SF: Social Function
SD: Standard deviation
SF-36: MOS 36-item Short-Form Health Survey
VT: Vitality
